# Assessing the Relationship Between Motor Anticipation and Cortical Excitability in Subacute Stroke Patients With Movement-Related Potentials

**DOI:** 10.3389/fneur.2018.00881

**Published:** 2018-10-17

**Authors:** Ling Chen, Yurong Mao, Minghui Ding, Le Li, Yan Leng, Jiangli Zhao, Zhiqin Xu, Dong Feng Huang, Wai Leung Ambrose Lo

**Affiliations:** ^1^Department of Rehabilitation Medicine, Guangdong Engineering and Technology Research Center for Rehabilitation Medicine and Translation, The First Affiliated Hospital, Sun Yat-sen University, Guangzhou, China; ^2^Department of Acupuncture and Moxibustion, The Secondary Medical College, Guangzhou University of Traditional Chinese Medicine, Guangzhou, China; ^3^Xinhua College of Sun Yat-sen University, Guangzhou, China

**Keywords:** stroke, subacute, motor anticipatory, cortical excitability, movement-related potential

## Abstract

**Background:** Stroke survivors may lack the cognitive ability to anticipate the required control for palmar grasp execution. The cortical mechanisms involved in motor anticipation of palmar grasp movement and its association with post-stroke hand function remains unknown.

**Aims:** To investigate the cognitive anticipation process during a palmar grasp task in subacute stroke survivors and to compare with healthy individuals. The association between cortical excitability and hand function was also explored.

**Methods:** Twenty-five participants with hemiparesis within 1–6 months after first unilateral stroke were recruited. Twenty-five matched healthy individuals were recruited as control. Contingent negative variation (CNV) was measured using electroencephalography recordings (EEG). Event related potentials were elicited by cue triggered hand movement paradigm. CNV onset time and amplitude between pre-cue and before movement execution were recorded.

**Results:** The differences in CNV onset time and peak amplitude were statistically significant between the subacute stroke and control groups, with patients showing earlier onset time with increased amplitudes. However, there was no statistically significant difference in CNV onset time and peak amplitude between lesioned and non-lesioned hemisphere in the subacute stroke group. Low to moderate linear associations were observed between cortical excitability and hand function.

**Conclusions:** The earlier CNV onset time and higher peak amplitude observed in the subacute stroke group suggest increased brain computational demand during palmar grasp task. The lack of difference in CNV amplitude between the lesioned and non-lesioned hemisphere within the subacute stroke group may suggest that the non-lesioned hemisphere plays a role in the motor anticipatory process. The moderate correlations suggested that hand function may be associated with cortical processing of motor anticipation.

## Introduction

### Cognitive process of movement anticipation in stroke patients

Stroke is among the leading causes of long-term disability worldwide (1). It is one of the most severe issues encountered by the aging population (2). Seventy-five percent of stroke survivors have motor dysfunction that affects body coordination and motor skill (3). Motion prediction is a key component of cognitive function and is a high-level function that affects motor control (4). Motor function recovery is often measured in terms of motor execution, with little consideration given to the high level cognitive processes that feeds into the actual motor response (5). To execute activities of daily living such as reach and grasp, the upper extremity must apply the correct force, move the precise range and accurately coordinate multiple limb segments (6–10). The cognitive ability to anticipate the required movement control is therefore fundamental to hand motor performance. Published literature indicates that stroke patients lack the anticipatory ability of upper limb movement that is associated with palmar grasp (11–13). The lack of ability to anticipate was evidenced in the suboptimal application of force by producing markedly increased grip forces during lifting, holding and moving a hand-held object in patients with acute stroke (14). Patients with chronic stroke demonstrated a slower response to adapt to the perturbing force and exhibited smaller aftereffects when the perturbing force was unexpectedly removed than healthy controls (11).

### Contingent negative variation

The electrophysiological processes associated with movement anticipation and the relationship between the electrophysiological changes and clinical impairment of hand function in stroke patients remain poorly understood (15). Contingent negative variation (CNV) is one of the electrophysiological substrates of motor anticipation. CNV was first reported by Walter et al. (16) as a slow-going, negative event-related potential (ERP) that reflects the cognitive processing (5). It refers to the sustained negativity that develops after the pre-cue (S1) and before the imperative stimulus (S2) that requires a response. If the interval between S1 and S2 is >2 s, CNV can be distinguished into initial CNV and late CNV (17). The late CNV that occurs before S2 is assumed to be the composition of a readiness potential and stimulus preceding negativity (18). The amplitude of late CNV is also modulated by the amount of advanced information provided by the pre-cue (19–21), which usually shows up as an increase in late CNV. Late CNV tends to increase when people perform intention-based action with advanced information provided by pre-cue (21–23). This is because pre-cue information enables people to have sufficient anticipation for the upcoming action (24). Source analysis suggests that there are multiple sources of the generation of late CNV. These include the cortical and subcortical generators of the (1) anterior cingulate cortex, (2) supplementary motor area, and (3) primary motor area, in particular the inferior parietal cortex (19, 25, 26). CNV is suggested to be an index of anticipation because it reflects the action preparation process (21, 27). Therefore, the CNV amplitude and latency represent cognitive processes of anticipation and movement preparation (28–30). Early literature reported that the time between the onset of brain potential recorded by electroencephalographic (EEG) and movement onset recorded with electromyography (EMG) were indicative of the preparation time for the required action (31). Studies of healthy individuals also indicated that non-dominant hand movement has an earlier onset of cortical potential when compared with the dominant hand movement. Thus, there is evidence to indicate that the longer anticipatory phase reflects the increase in computational demand when executing learned movement (32). In people with chronic stroke, CNV is significantly enhanced at the midline region with markedly slower response time during affected hand preparation (5). The increased amplitude of the midline region CNV correlates with a greater response-priming effect. Enlarged CNV amplitudes during intention-based actions are expected in people with stroke as they need to have increased cognitive preparation for the anticipation process when compared with healthy individuals. Similar findings were also reported in chronic stroke patients who had no residual paretic hand movements (33). Simultaneous recording of EEG and EMG indicated that early onset of cortical potential precede affected rather than unaffected hand movement.

To date, the cognitive process involved in movement anticipation during a palmar grasp task with advance pre-cuing in the subacute stroke population has not been studied using the CNV approach. Understanding the cortical regions that are involved during grasping preparation in the early stages of stroke can provide new insight into the neural plasticity mechanism for grasp motion recovery. The aim of the present study was to investigate the difference in the cognitive anticipation process between the lesioned and non-lesioned hemisphere in subacute stroke survivors and to compare the difference in the anticipation process with healthy individuals. This study hypothesized that subacute stroke patients demonstrated a statistically significant difference in CNV amplitude during intention-based action between the lesioned and non-lesioned side and that this differed significantly from healthy individuals. The study also investigated if the difference in the electrophysiological process was associated with a functional level as measured by the Action Research Arm Test (ARAT).

## Methods

### Participants

Twenty-five participants (thirteen males and 12 females; aged 55.2 ± 8.4 years) with subacute stroke were recruited. Twenty-five age and gender matched healthy individuals (thirteen males and 12 females; aged 53.8 ± 7.9 years) were recruited as controls. The functional level of the participants with subacute stroke was assessed by the ARAT (34), which consists of a total of 19 tests of arm motor function including grasp, hold, pinch and gross motor movement. Each test is allocated an ordinal value of 0, 1, 2, or 3, with higher values indicating better arm motor status. The total ARAT score is the sum of the 19 tests, and the maximum score of the scale is 57 (35). All recruited participants were right hand dominant as assessed by the Edinburgh handedness inventory (36). Table 1 gives a summary of the demographic data of the sample population.

**Table 1:** A summary of the demographic data of the included subjects in the stroke group.

	**Age**	**Onset time**	**NIHSS**	**ARAT score**
Mean	55.2	59.1	5.4	15.2
SD	8.4	24.9	3.0	12.6

The inclusion criteria for the subacute stroke cohort were as follows: (1) hemiparesis resulting from a unilateral subcortical lesion of the first occurrence of a stroke; (2) within 1–6 months of stroke occurrence; (3) magnetic resonance imaging (MRI) or computed tomography (CT) confirmed stroke; (4) age between 40 and 80 years old; (5) moderate to severe motor deficit of the affected upper limbs, having at least 10° of finger flexion and extension; (6) be able to sit at least 30 min without assistance; and (7) no severe cognitive impairment (Mini Mental State Examination >21) (37). None of the participants had any prior brain computer interface (BCI) experience. The exclusion criteria were as follows: (1) lacunar infarction; (2) massive cerebral infarction; (3) cerebellum or brainstem lesion; (4) open hand wound or hand deformity; and (5) visual field deficits. All of the healthy individuals had no history of psychiatric/neurological disease or musculoskeletal disorder.

### Ethics considerations

The study was approved by the Ethical Committee of The First Affiliated Hospital of Sun Yat-sen University. Data collection took place in the Department of Rehabilitation Medicine, The First Affiliated Hospital of Sun Yat-sen University. All of the participants were provided with a comprehensive explanation of the experimental protocol. They were given an information sheet about the study and encouraged to ask questions. Written informed consent was obtained from all participants.

### Experimental protocol

Data recording took place in an electrically shielded brain function laboratory. All participants were seated in front of a table. They were asked to place their shoulders at between 0 and 10° flexion, their forearms rested on the table with elbows flexed to 130°, and their wrists were oriented in a neutral position so that opening and closing of the hand occurred in the horizontal plane. Participants were asked to fix their gaze in the middle of the screen straight ahead, avoid eye movement and focus attention on task performance. All participants had one practice session before the ERP recording began.

#### Experimental task and procedure

All the participants first undertook 5 to 10 min of training to familiarize themselves with the entire procedure before the ERP recording began. At the beginning of the experiment, the introduction was displayed on the screen. The instructions given were as follows:

“*Welcome to this experiment. During the experiment, you will see a fixated white cross appear on the screen to remind you to pay attention to the task. Then, you will see a picture of either a left hand grasp or a right hand grasp displayed on the screen accompanied by a sound to indicate a left or right hand grasp. At this stage, please keep both hands still. The screen will then turn to a grey window. During the grey window, please do a self-paced voluntary palmar grasp according to the previous cue. Throughout the experiment, please refrain from blinking and body movements, particularly during the opening and closing of the hands.”*

**Figure 1:**
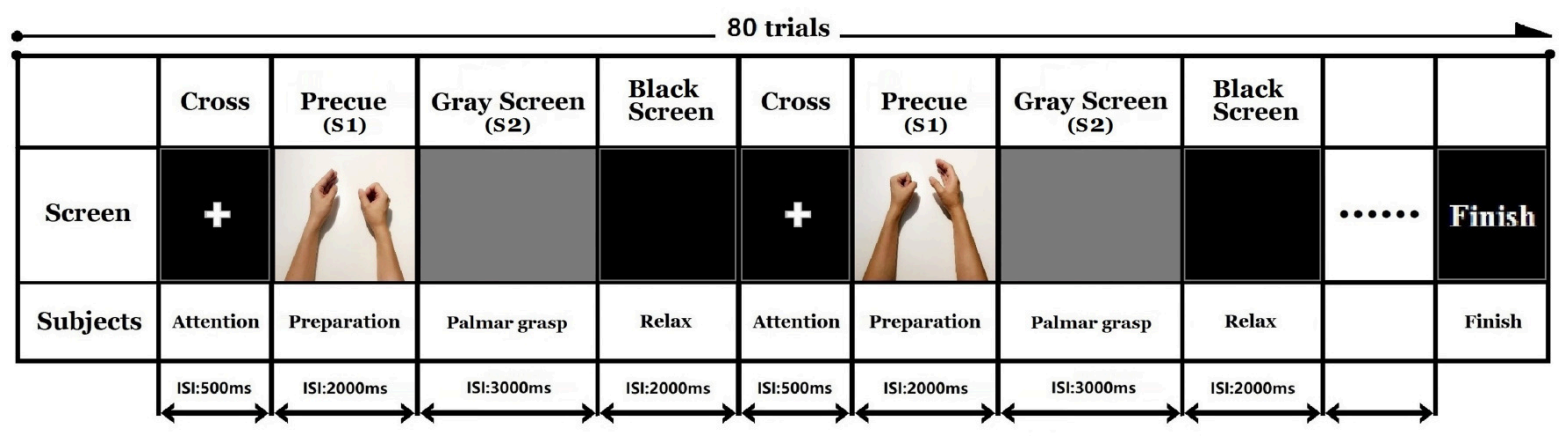
Experimental trial sequence. *Key*: ISI, inter stimulus interval.

After understanding the displayed instructions, participants pressed the space bar to start the experiment. Participants first performed a modified audio-visual task that resembled a palmar grasp movement. The fixated white cross appeared on the screen for 500 ms to remind participants to pay attention to the task. Simultaneous visual and auditory imperative cues (S1) were given for 2,000 ms. A picture was displayed on the screen accompanied by a sound to indicate a right or left side palmar grasp. During S1, the participant was required to acquire the visual and auditory cues and judge the palmar grasp task. The screen then turned to a gray reaction window (S2) for 3,000 ms. The participants performed a self-paced voluntary palmar grasp during the S2 window. The experiment consisted of 40 trials for each hand, giving a total of 80 trials. The order of the trials was randomized in each experiment. The inter trial resting interval was marked by a dark screen that was displayed for 2,000 ms. The sequence of events in the trials is illustrated in Figure 1. All the participants were asked to avoid blinking and making compensatory/additional movements during the opening and closing of the hand.

#### EEG signal acquisition

The EEG data of each participant was recorded by a 32-channel QuickAmp amplifier and Ag/AgCl scalp electrodes (BrainProducts, Germany). The electrodes were positioned in accordance with the international 10–20 system (FP1, FP2, F3, Fz, F4, FC5, FC3, FC1, FCz, FC2, FC4, FC6, C5, C3, C1, Cz, C2, C4, C6, CP5, CP3, CP1, CPz, CP2, CP4, CP6, P3, POz, P4, POz, O1, O2; reference: FCz, ground: AFz). Data were recorded in DC mode with a sampling rate of 1,000 Hz. Electrodes were filled properly with conductive gel to maintain the impedance below 5 kΩ to ensure good quality recording.

**Figure 2:**
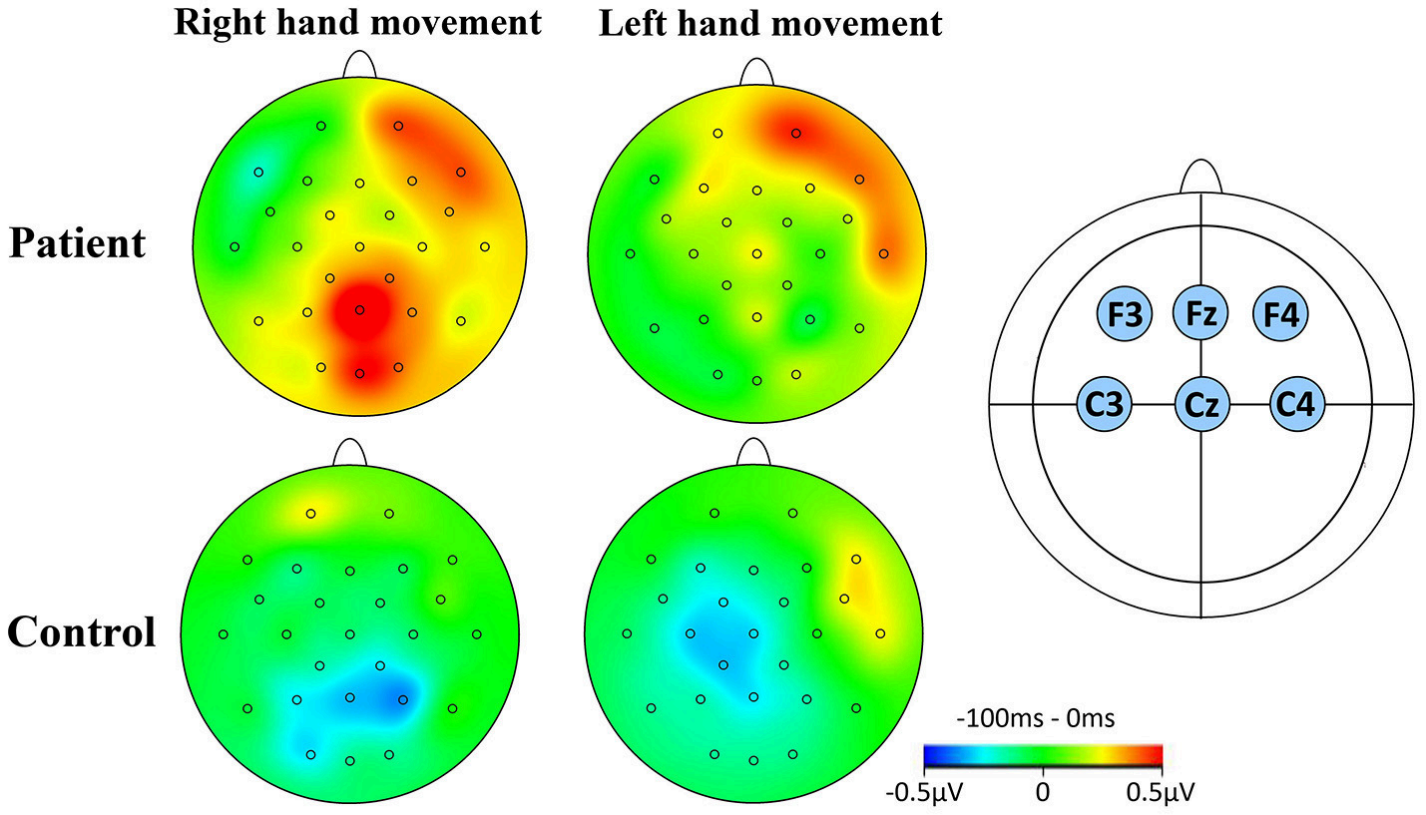
CNV topographical distributions. **(A)** The window for the mapping was −100 to 0 ms relative to S1. Red contour lines indicate positive activity, blue contour lines indicate negative activity. The greyscale fill between the contour lines indicates amount of activation (positive or negative). The small circles on the topographies indicate the electrodes sites. The rows indicate the two groups studied during RHM and LHM. **(B)** The location of the six electrodes of interest.

For CNV amplitude analysis, topographic mapping was performed using six electrodes of interest. These electrodes were (i) F3 and C3 (left hemisphere), (ii) F4 and C4 (right hemisphere), and (iii) Fz and Cz (midline region). Figure 2 illustrates the topographic maps of participants when executing left or right hand movement tasks.

#### EEG signal processing

EEG signal analysis was performed using the Brain Vision Analyzer signal processing software 2.0 (BrainProducts, Germany). During the EEG pre-processing, the average potential of the bilateral mastoid was used as a new reference. Eye movement artifacts were removed through the Ocular Correction Independent Component Analysis (ICA) (38). The removal of eye movement artifacts is a standard operating procedure when analyzing EEG signals. The number of trials or the quality of data were not affected. Inspection of the raw data was conducted with a 50 Hz notch filter and a 0.1–30 Hz bandpass filter. The EEG data were segmented into epochs of 500 ms pre to 3,000 ms post-aligned to the cue (S1) to acquire stimulus-locked ERPs, and the baseline was corrected to the first 200 ms of the epochs, which was the 200 ms time window before S1 onset. For each epoch, the late CNV onset time was calculated as the last time the signal crossed the zero line before the rise of the CNV (17) (see **Figure 4**), and the peak amplitude was calculated from 1,000 to 2,000 ms since this time window contained the maximal variation of the CNV potential. CNV onset time and peak amplitude features were extracted.

In this study, the left and right hemispheres of subacute stroke participants were labeled as “lesioned” and “non-lesioned.” The left and right sided lateralised scalp sites were flipped in the participants with the right hemispheric lesion, (e.g., C3 and F3 for the right lesions and C4 and F4 for the left lesion) (33) to enable statistical comparisons between the lesioned and non-lesioned hemisphere. Thus, the “left” hemisphere was always the lesioned hemisphere in the subacute stroke group.

### Statistical analysis

All statistical analyses were performed using SPSS Statistic 21. Descriptive statistics were conducted to describe the sample population. CNV onset time and peak amplitudes were analyzed with a mixed model ANOVA analysis that incorporated the within-subjects factors TASK (Left hand movement and Right hand movement) and the LATERALITY (midline, contralateral, and ipsilateral regions). The between-subject factor of GROUP was used to compare the subacute stroke group to the matched healthy control group (stroke vs. control) during right-hand movement (RHM) and left-hand movement (LHM) and to compare the lesioned and non-lesioned hemisphere within the subacute stroke group during RHM and LHM. The Greenhouse-Geisser method were used to adjust the *p*-value and degrees of freedom when the assumption of sphericity was not met. Separate ANOVAs were calculated for each level of LATERALITY, using the same mixed model ANOVA format. To further investigate the differences between these groups, subsequent independent sample *t*-tests were performed. The significance level for all statistical analysis was set at 0.05. Bonferroni-adjusted significance tests were performed to correct the *p*-values of electrodes for multiple comparisons. Thus, the corrected significance level for LATERALITY was α = 0.05 ÷ 6 = 0.008. Spearman's rank correlation analysis was performed to investigate the association between the ARAT and electrophysiological measures. The interpretations of Spearman's rank correlation coefficient were as follow: “slight” (0.0–0.2); “low” (0.2–0.4); “moderate” (0.5–0.7); “high” (0.8–0.9) (39).

## Results

### Onset time

**Figure 3:**
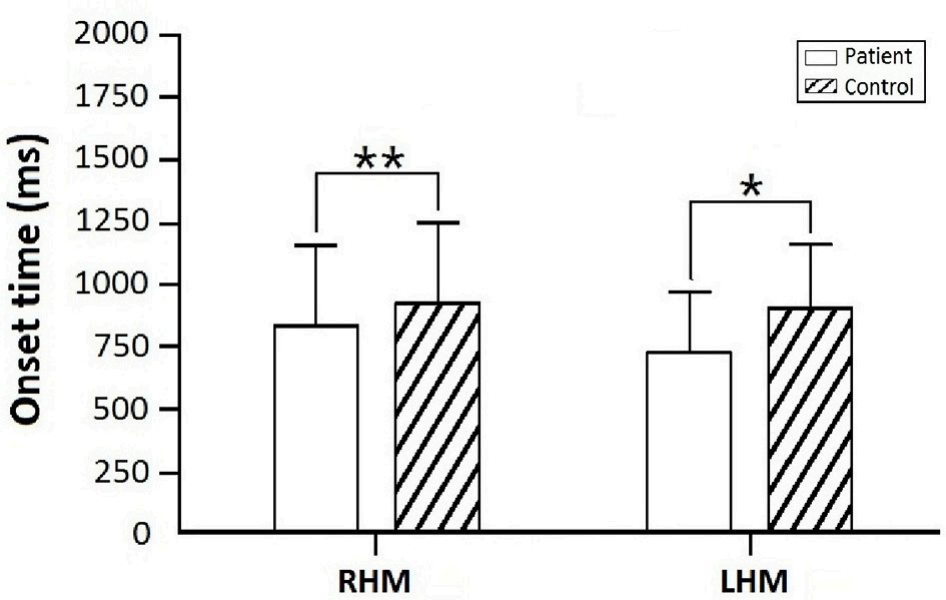
Onset time for the subacute stroke group in comparison to control group. Asterisks indicate significant differences within RHM and LHM (^**^p < 0.01; ^*^p < 0.05).

The mixed model ANOVA analysis indicated a significant main effect of TASK (RHM, LHM) on the CNV onset time [*F*_(1, 48)_ = 2.869, *p* = 0.028]. CNV onset time for patients with a subacute stroke was earlier than that of the control group both during RHM (*p* = 0.015) and LHM (*p* = 0.002). In addition, the difference was not statistically significant in CNV onset time between RHM (*p* = 0.323) and LHM (*p* = 0.202) within the subacute stroke group. Figure 3 illustrates the onset time for patients compared with controls.

### Peak amplitude

#### Analysis between subacute stroke group and control group

The mixed model ANOVA analysis on the CNV peak amplitude between the subacute stroke group and control group during RHM and LHM revealed a significant TASK × GROUP × LATERALITY interaction [*F*_(5, 240)_ = 5.635, *p* < 0.001]. Furthermore, comparisons between the subacute stroke group and their match control group indicated a significant main effect of TASK for CNV peak amplitude [*F*_(2.619, 125.713)_ = 3.390, *p* = 0.025]. Thus, separate ANOVAs were calculated for each level of TASK.

##### Comparison between subacute stroke group and control group within RHM condition

**Figure 4:**
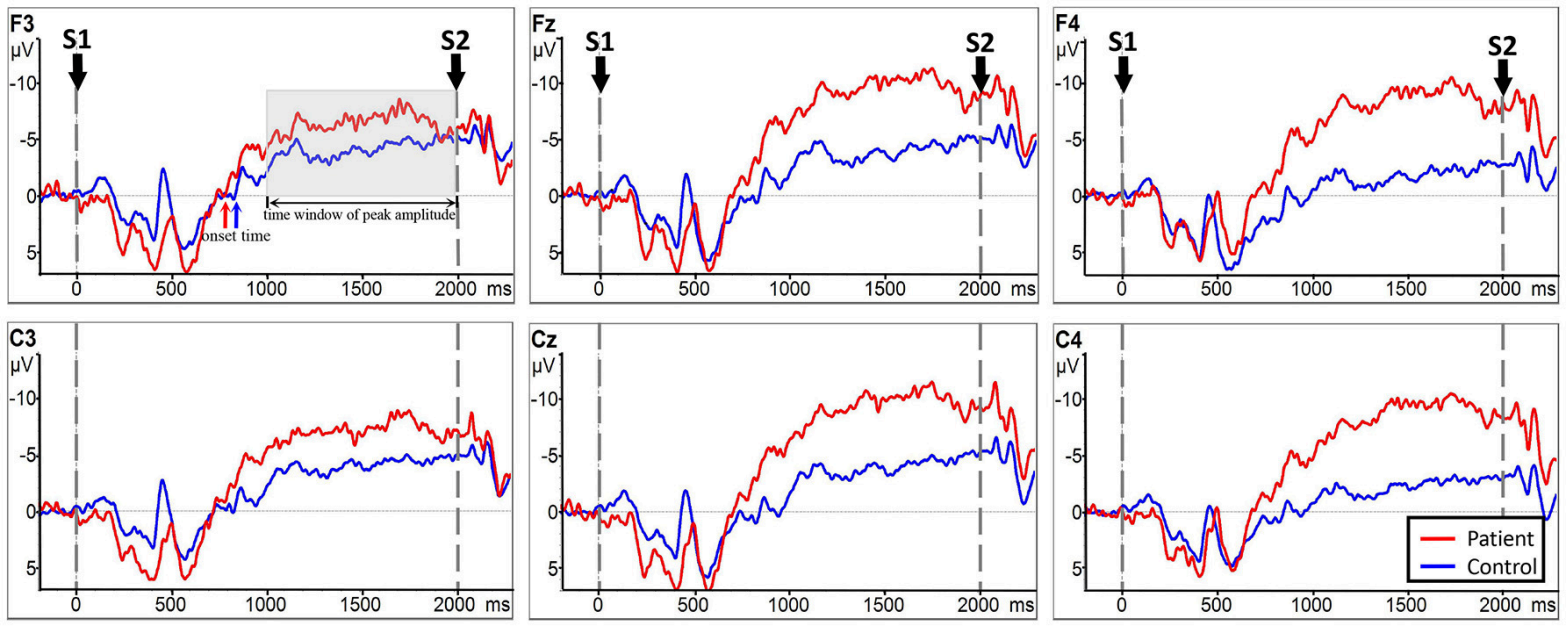
CNV amplitudes for the subacute stroke group (red line) and control group (blue line) during RHM. The Y-axis represents CNV waveform (μ*V*) and the X-axis represents time (ms). Negative is plotted upwards. Baseline is the first 200 ms of the epoch, i.e., 200 ms before S1 onset. The 0 ms point is S1 onset. Red/blue arrows represent the onset of the late CNV in patient group/control group. The gray time window indicated the time window to detect the peak amplitude.

Analysis between the two groups during RHM indicated no significant LATERALITY × GROUP interaction [*F*_(2.980, 71.520)_ = 1.605, *p* = 0.196]. Therefore, LATERALITY and GROUP were tested for individual effects. Significant GROUP effect [*F*_(1, 24)_ = 9.379, *p* = 0.002] and LATERALITY effect [*F*_(2.738, 262.837)_ = 4.364, *p* = 0.001] were observed. Then, subsequent independent sample *t*-tests revealed significantly larger CNV amplitudes in the subacute stroke group at midline (Fz, Cz), ipsilateral (F4, C4), and contralateral (C3, F3) electrodes than the control group when conducting right hand movement. Figure 4 gives the graphical representations of the CNV amplitudes during RHM. Table 2 illustrates the comparison of CNV peak amplitude between the two groups within RHM condition.

**Table 2:** Comparison of CNV peak amplitude between subacute stroke group and control group within RHM condition.

**Electrodes**	**Stroke group**	**Control group**	***p*-value**
C3	−14.057 (16.037)	−7.830 (8.010)	0.005^**^
Cz	−14.112 (17.994)	−7.055 (9.056)	0.001^**^
C4	−11.713 (15.935)	−7.173 (6.047)	0.005^**^
F3	−11.942 (16.422)	−8.616 (6.943)	0.009^**^
Fz	−12.387 (15.454)	−7.010 (9.533)	0.001^**^
F4	−11.063 (15.973)	−6.450 (7.190)	0.006^**^

Asterisks indicate significant differences (

** *p < 0.01)*.

##### Comparison between subacute stroke group and control group within LHM condition

**Figure 5:**
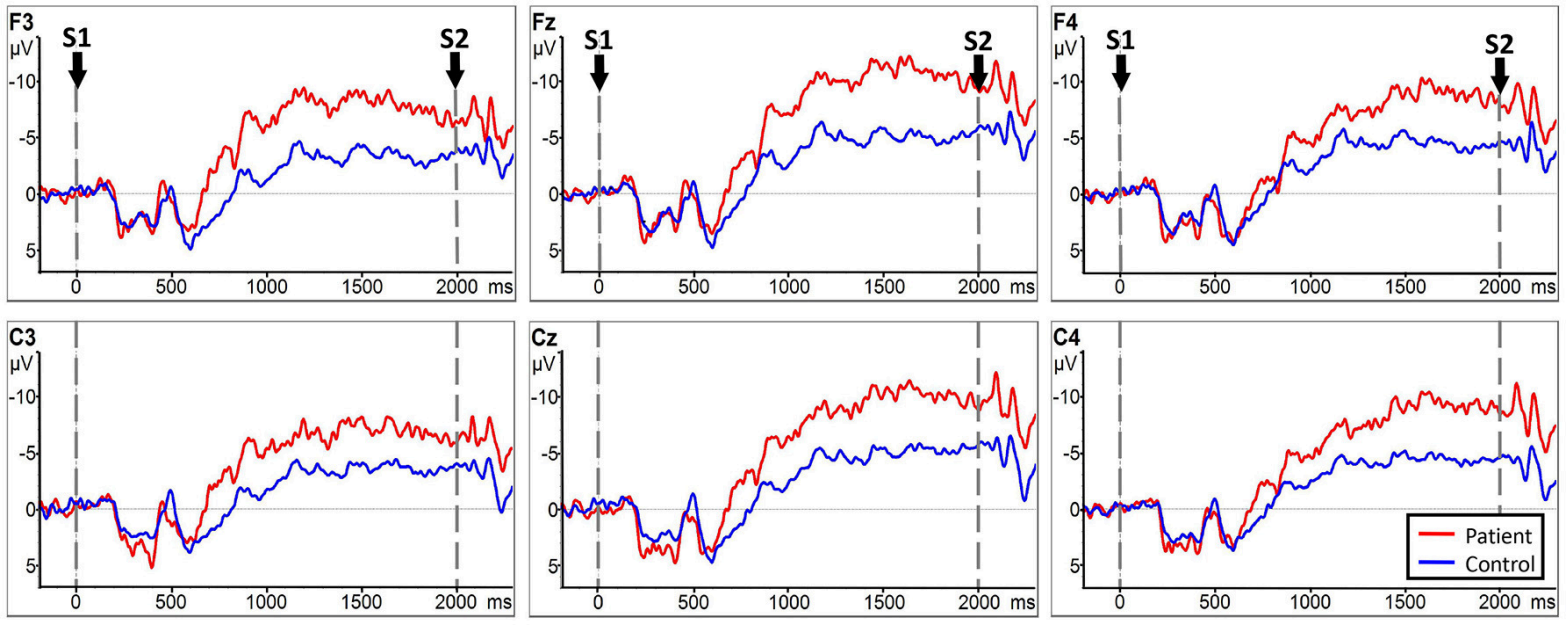
CNV amplitudes for the subject stroke group (red line) and control group (blue line) during LHM. The Y-axis represents CNV waveform (μ*V*) and the X-axis represents time (ms). Negative is plotted upwards. Baseline is the first 200 ms of the epoch, i.e., 200 ms before S1 onset. The 0 ms point is S1 onset.

No significant LATERALITY × GROUP interaction [*F*_(2.078, 49.883)_ = 0.905, *p* = 0.415] was observed in CNV amplitude between the subacute stroke group and the control group. Therefore, LATERALITY and GROUP were tested for individual effects. Analysis showed significant GROUP effect [*F*_(1, 24)_ = 14.47, *p* = 0.001] and LATERALITY effect [*F*_(2.920, 70.073)_ = 7.185, *p* < 0.001]. Further investigation of these effects revealed that there was a significantly larger CNV amplitude in the subacute stroke group at midline (Fz, Cz), contralateral (F4, C4), and ipsilateral (F3, C3) regions than the control group when conducting right hand movement. Figure 5 gives the graphical representations of CNV amplitudes for the patient and control groups during LHM. Table 3 shows the comparison of CNV peak amplitudes between subacute stroke group and control group within LHM condition.

**Table 3:** Comparison of CNV peak amplitude between subacute stroke and control group within LHM condition.

**Electrodes**	**Stroke group**	**Control group**	***p*-value**
C3	−12.250 (14.180)	−7.826 (3.529)	0.005^**^
Cz	−15.453 (11.262)	−7.727 (6.187)	0.001^**^
C4	−14.165 (14.923)	−8.566 (5.644)	0.001^**^
F3	−12.932 (11.318)	−6.869 (5.986)	0.007^**^
Fz	−15.800 (11.153)	−8.587 (6.364)	0.001^**^
F4	−12.017 (11.461)	−7.973 (5.029)	0.001^**^

Asterisks indicate significant differences (

** *p < 0.01)*.

#### Comparison between RHM and LHM within the subacute stroke group

**Figure 6:**
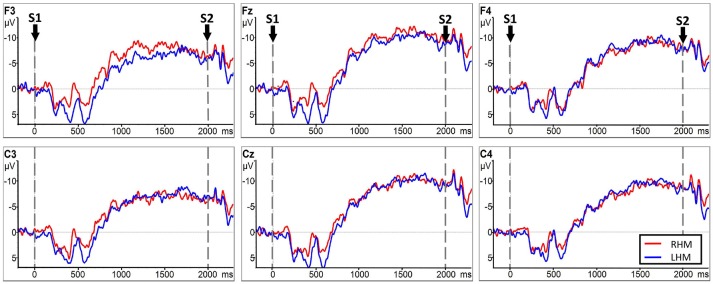
CNV amplitudes for the subacute stroke group group during RHM (red line) and LHM (blue line). The Y-axis represents CNV waveform (μ*V*) and the X-axis represents time (ms). Negative is plotted upwards. Baseline is the first 200 ms of the epoch, i.e., 200 ms before S1 onset. The 0 ms point is S1 onset.

Analysis indicated no significant LATERALITY × GROUP interaction [*F*_(2.153, 51.669)_ = 0.856, *p* = 0.438]. Further analysis showed no statistically significant main effect of GROUP [*F*_(1, 24)_ = 0.100, *p* = 0.755] or LATERALITY [*F*_(2.372, 56.938)_ = 1.209, *p* = 0.051] on CNV amplitude between RHM and LHM within the subacute stroke group. Thus, there was no statistically significant difference in CNV amplitude between RHM and LHM in the subacute stroke group. Figure 6 shows the EEG traces recorded at each electrode during LHM and RHM in the subacute stroke group.

When comparing laterality factors (contralateral, midline, ipsilateral) within the TASK movement condition (RHM and LHM), there was no statistically significant hemisphere LATERALITY effect on CNV amplitude during RHM [*F*_(5, 65.715)_ = 2.015, *p* = 0.126].

During LHM, a significantly main effect of LATERALITY on CNV amplitude was observed [*F*_(1.944, 46.663)_ = 3.820, *p* = 0.030]. Further investigation of the hemisphere LATERALITY effect revealed that midline electrodes (Cz) had a higher CNV amplitude than contralateral (C4) electrodes (*p* = 0.007).

### Correlations between CNV peak amplitude with ARAT in the subacute stroke group

**Figure 7:**
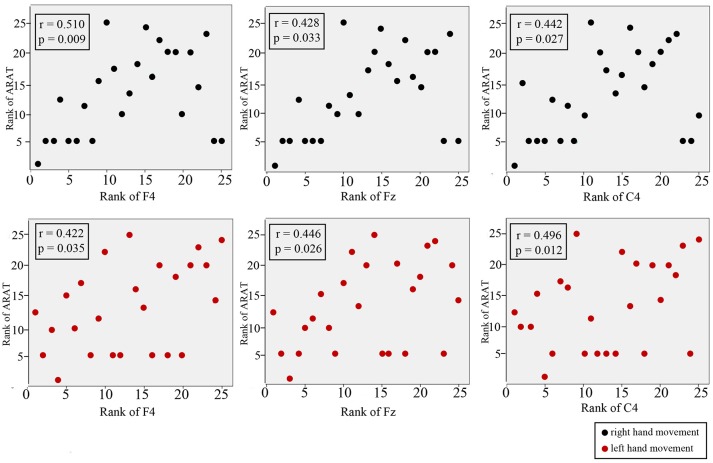
Correlation between CNV amplitude with ARAT in stroke participants. The Y-axis represents the rank of ARAT and the X-axis represents the rank of CNV. CNV recorded at midline, front-contralesional/front-ipsilateral side, and contralesional/ipsilateral side shown for RHM (black circles) and LHM (red circles).

Figure 7 shows the results of Spearman's rank correlation coefficient analysis between the CNV peak amplitude observed at different regions and the ARAT within the subacute stroke group. During RHM, significant correlations were observed in the front-ipsilateral side (*r* = 0.510, *p* = 0.009), midline (*r* = 0.428, *p* = 0.033), and ipsilateral side (*r* = 0.442, *p* = 0.027). During the LHM condition, a significant correlation was observed in the front-contralesional side (*r* = 0.496, *p* = 0.012), midline (*r* = 0.446, *p* = 0.026), and contralesional side (*r* = 0.496, *p* = 0.012).

## Discussion

The primary aim of this study was to investigate the difference in the cognitive anticipation process between the lesioned and non-lesioned hemisphere in subacute stroke survivors and to compared those with healthy individuals. The association between the electrophysiological process and the functional level as measured by the ARAT in the stroke group was also investigated.

### Onset of CNV

Results of this present study are consistent with existing literature reporting extended anticipation time. We found that this occurred in the subacute stroke population when compared with the control group, as evidenced by the statistically significant early CNV onset time during left hand and right hand movement. This finding is consistent with a previous investigation that reported a significantly earlier onset time in the paretic hand movement than in the non-paretic hand movement in people with chronic stroke using movement-related slow cortical potentials (SCP) (33). This supports the theory that extended planning time is needed for the brain to start exciting the motor network that leads to movement execution of the paretic arm. The results of this present study are consistent with existing literature suggesting that extended anticipation (planning) time is present within the subacute stroke population when compared with a control group. This study however did not observe any difference in CNV onset time between the lesioned and non-lesioned side during LHM and RHM. This is contrary to previous studies that reported a significant difference in CNV onset time between paretic and non-paretic hand movement (5, 33). The different findings between the two studies are likely related to the difference in experimental design. In the study by Deecke et al. (40), slow cortical potential measurement incorporated both anticipation of an upcoming movement as well as the motor potential occurring at the time of movement execution. Thus, SCP did not only reflect the cognitive processing behavior but also the additional time required for motor network excitation. The study by Dean et al. (5), recorded CNV onset from S2 (response cue) until feedback (task execution), whereas this study measured from S1 (pre-cue) until S2 without task execution. Thus, the potentials reported by Dean et al., did not only reflect the anticipation but also the potentials during movement. The findings in this study demonstrated for the first time that the motion anticipation phase alone is earlier in subacute stroke participants than healthy participants. Furthermore, advanced motor anticipation occurred not only in the paretic hand but also in the non-paretic hand, as evidenced by the lack of difference in CNV between the lesioned and non-lesioned hemisphere.

### CNV amplitude related to motor expectancy

This study evaluated motor anticipation with CNV elicited by a stimulus-locked open/close hand movement paradigm. People with motor impairment are expected to have a larger peak amplitude that reflects the increase in the brain's computational demand to execute a movement (41). The study observed a significantly larger peak amplitude of CNV during RHM (affected hand) in the stroke group over the contralateral (C3), midline (Fz and Cz), and ipsilateral (C4) regions than that in the matched control group. This finding is consistent with published literature and supports the notion of an increase in psychological anticipation during the affected hand movement. This study also observed an increase in the activity pattern at contralateral (C4), midline (Fz and Cz), and ipsilateral (C3) locations during the LHM (unaffected hand) in the stroke group when compared with the control group. This suggests that increased psychological anticipation activity also occurs during unaffected hand movement. This higher activity level may be related to the compensatory over activation in the non-lesioned hemisphere and the lack of cross hemispheric inhibition from the lesioned to non-lesioned hemisphere.

Within the stroke group, extensive anticipation activation was observed at the frontal, contralateral, midline, and ipsilateral regions during the affected hand movement. No difference was found between the contralateral (C3) and ipsilateral (C4) side, with a significantly larger peak amplitude observed at the midline (Cz) than ipsilateral (C4) side. This suggests that there is no definitive hemisphere laterality and that most of the anticipation activity originates from the midline region during affected palmar movement. This is consistent with studies that reported increased effort in response to the inability to control the process in the hemisphere (42–44). These results did not demonstrate the effect a pattern of larger CNV amplitude over the contralateral region, as reported in previous research in the chronic stroke population (5, 19, 45, 46). The difference in findings may be related to the chronicity of the sample population. Cortical map expansion has been repeatedly reported in animal (47) and human experiments (48) after cortical damage. The expansion happens during the early stages of a stroke due to neuroplasticity and up to 8 weeks after training had stopped (49). Once the sequence of the motor task is learned, the size of the mapping representation returns to its original size (50). The lack of a significant difference in CNV amplitude between the left and right hemisphere may suggest that the subacute stroke patients are still going through the learning process to regain the palmar grasp movement. Thus, cortical expansion is occurring during this stage due to the neuroplasticity process, and a significantly larger CNV amplitude is generated along with bilateral changes in the adaptive compensation function of the brain (41). This theory is given further support from early literature that has repeatedly shown that movement of the affected hand is associated with increased bilateral activation of sensorimotor cortex (51–54). The lack of difference in the peak CNV amplitude may also be related to the incongruous over activation that occurs in the unaffected hemisphere or to an imbalance in inter hemispheric inhibition (55–59).

### Correlation between electrophysiological measures and clinical scale

A higher ARAT score is indicative of higher motor function of the paretic hand (60). Though it appeared that a better ARAT performance correlated with a smaller CNV peak amplitude, it is not possible to draw a firm conclusion because there is insufficient data available from the high and low CNV amplitude comparison. This study observed the strongest significant positive correlation at the front-ipsilateral side, which suggests that motor function may be represented by neural pathway modulation that is responsible for motor function. The significant moderate correlations suggested that hand function may be associated with cortical excitability. The correlation level observed in this study may be affected by the spread of the data. A known limitation of the correlation coefficient is that it is affected by the spread of the data, i.e., the bigger the data range, the higher the chance of a high correlation (61).

### Limitations

There are some important points regarding the limitations of this study. One limitation was that we recruited a relatively small sample size of subacute stroke participants and matched healthy controls (both groups *n* = 25). Future research will be needed both to enroll chronic upper limb hemiparesis stroke subjects and enlarge the sample size to explore the effects of neurophysiologic mechanisms identified in the present study. Second, this study focused on participants with moderate to severe motor deficits of the affected upper limbs. Further research is suggested to investigate whether stroke patients with mild motor deficits of the affected upper limbs can also elicit electrophysiological changes of motor anticipation. Third, the ERP has the characteristic of temporal resolution of the millisecond, which can accurately capture extremely weak signals from the brain and observe the cortical excitability of motor anticipation in real time (62). To explore further insight into the strict frequency locked cortical excitability of motor anticipation, event related synchronization technology is recommended for further research. The flipping of scalp sites may be considered a limitation. However, it is not uncommon in published literature that involves ERP or fMRI to flip the scalp site in an attempt to increase statistical power. In addition, the preliminary analysis of the present results indicated there was no statistically significant difference in CNV onset time or amplitude between patients with a left and right hemispheric lesion. Thus, the inverted electrodes in right-hemisphere lesioned patients were unlikely to alter the current findings.

## Conclusions

This study investigated the electrophysiological changes of motor anticipation during a palmar grasp task in subacute stroke survivors. The earlier CNV onset time and the higher peak amplitude are indicative of increased brain computational demand during palmar grasp task post-stroke. These are indicative of an increase in brain computational demand during the palmar grasp task. The lack of difference in CNV amplitude between the lesioned and non-lesioned hemisphere in the subacute stroke group may suggest that the non-lesioned hemisphere may play a role in the anticipatory process. Further investigation is required to understand the role of the non-lesioned side in movement recovery and the impact of intervention on electrophysiological changes.

## Author contributions

All authors have read and approved the final manuscript. All authors meet the four primary ICMJE criteria for authorship. In addition, all authors have been actively involved in the study in different capacities: LC designed the study and conducted all stages of the study including data collection, analysis, interpretation, and drafting of the manuscript. YM participated in the design of research protocol and interpreted the results. YL participated in the participants recruitment. MD participated in the recruitment and data analysis. LL revised the manuscript interpreted the data. JZ and ZX assessed the motor deficit of the affected upper limbs with Research Arm Test (ARAT). DH and WL managed the trial. WL analyzed and interpreted the data and revised the manuscript.

### Conflict of interest statement

The authors declare that the research was conducted in the absence of any commercial or financial relationships that could be construed as a potential conflict of interest.
